# Disentangling the evolution of electrons and holes in photoexcited ZnO nanoparticles

**DOI:** 10.1063/4.0000204

**Published:** 2023-11-03

**Authors:** Christopher J. Milne, Natalia Nagornova, Thomas Pope, Hui-Yuan Chen, Thomas Rossi, Jakub Szlachetko, Wojciech Gawelda, Alexander Britz, Tim B. van Driel, Leonardo Sala, Simon Ebner, Tetsuo Katayama, Stephen H. Southworth, Gilles Doumy, Anne Marie March, C. Stefan Lehmann, Melanie Mucke, Denys Iablonskyi, Yoshiaki Kumagai, Gregor Knopp, Koji Motomura, Tadashi Togashi, Shigeki Owada, Makina Yabashi, Martin M. Nielsen, Marek Pajek, Kiyoshi Ueda, Rafael Abela, Thomas J. Penfold, Majed Chergui

**Affiliations:** 1 European XFEL, D-22761 Hamburg, Germany; 2 SwissFEL, Paul Scherrer Institut, 5232 Villigen-PSI, Switzerland; 3 Lausanne Centre for Ultrafast Science (LACUS), ISIC, FSB, Ecole Polytechnique Fédérale de Lausanne, CH-1015 Lausanne, Switzerland; 4 Chemistry—School of Natural and Environmental Sciences, Newcastle University, Newcastle upon Tyne, NE1 7RU, United Kingdom; 5 Faculty of Physics, Adam Mickiewicz University, Uniwersytetu Poznańskiego 2, Poznań, 61-614, Poland; 6 IMDEA Nanoscience Institute, Calle Faraday 9, Campus Cantoblanco, 28049 Madrid, Spain; 7 Departamento de Química, Universidad Autónoma de Madrid, Campus Cantoblanco, 28047 Madrid, Spain; 8 The Hamburg Centre for Ultrafast Imaging, Luruper Chaussee 149, 22761 Hamburg, Germany; 9 Department of Physics, Technical University of Denmark, 2800 Kongens Lyngby, Denmark; 10 Japan Synchrotron Radiation Research Institute (JASRI), Kouto 1-1-1, Sayo, Hyogo 679-5198, Japan; 11 RIKEN, SPring-8 Center, Kouto 1-1-1, Sayo, Hyogo 679-5148, Japan; 12 Argonne National Laboratory, 9700 S. Cass Ave., Argonne, Illinois 60439, USA; 13 Advanced Research Center for Nanolithography (ARCNL), Science Park 106, 1098 XG Amsterdam, Netherlands; 14 Department of Physics and Astronomy, Uppsala University, 751 20 Uppsala, Sweden; 15 Institute of Multidisciplinary Research for Advanced Materials, Tohoku University, Sendai 980-8577, Japan

## Abstract

The evolution of charge carriers in photoexcited room temperature ZnO nanoparticles in solution is investigated using ultrafast ultraviolet photoluminescence spectroscopy, ultrafast Zn K-edge absorption spectroscopy, and *ab initio* molecular dynamics (MD) simulations. The photoluminescence is excited at 4.66 eV, well above the band edge, and shows that electron cooling in the conduction band and exciton formation occur in <500 fs, in excellent agreement with theoretical predictions. The x-ray absorption measurements, obtained upon excitation close to the band edge at 3.49 eV, are sensitive to the migration and trapping of holes. They reveal that the 2 ps transient largely reproduces the previously reported transient obtained at 100 ps time delay in synchrotron studies. In addition, the x-ray absorption signal is found to rise in ∼1.4 ps, which we attribute to the diffusion of holes through the lattice prior to their trapping at singly charged oxygen vacancies. Indeed, the MD simulations show that impulsive trapping of holes induces an ultrafast expansion of the cage of Zn atoms in <200 fs, followed by an oscillatory response at a frequency of ∼100 cm^−1^, which corresponds to a phonon mode of the system involving the Zn sub-lattice.

## I. INTRODUCTION

Transition metal oxides (TMO), such as titanium dioxide (TiO_2_) and zinc oxide (ZnO), are large-gap (>3.2 eV) semiconductors that have been attracting considerable interest in the past three decades or so, due to their remarkable optical properties, robustness under ambient conditions, abundance, and ease of preparation.^1,2^ This makes them potential candidates for photovoltaic and photocatalytic applications,^3–7^ detectors for high-energy radiation,^8^ transparent conductive oxides,^9^ lasing,^10^ pressure sensors with optical readout,^11,12^ etc. Their large band-gaps also offer the advantages of higher breakdown voltages, the ability to sustain large electric fields, lower noise generation, and high temperature and high-power operation.

These current and potential applications rely on the generation of charges and their subsequent evolution via electron–electron and electron–phonon scattering, diffusion through the lattice, thermalization and, eventually, localization either as self-trapped excitons (intrinsic trapping by electron–phonon coupling) or at defects (extrinsic trapping), followed by radiative and/or non-radiative electron–hole recombination. The initial events following photoexcitation take place at ultrashort time scales and need to be described in detail in order to reach optimal performances of the material for a specific application. This requires tools that can probe the evolution of charge carriers in real-time, are specific to both the valence (holes) and conduction (electrons) bands, and are, ideally, element-selective.

In the past 25 years or so, a large variety of ultrafast optical methods have been used to monitor the charge carrier dynamics in TMOs. In these experiments, a non-equilibrium distribution of electrons and holes is created upon above bandgap excitation, and the ultrafast (femtoseconds to picoseconds) evolution of charge carriers is monitored using different probes from the terahertz (THz) to the ultraviolet (UV) and visible spectral range.^13–22^ These probes are generally tuned to the intra-band transitions and, therefore, monitor the free carrier response, which does neither always distinguish between the electron and hole responses nor provides an unambiguous identification of trapping. Deep-UV probing of the inter-band transitions has also been implemented, as it can, in principle, distinguish between the dynamics occurring in the valence band (VB) and the conduction band (CB).^22–27^ However, the TA signal in this case is sensitive to the joint density-of-states (DOS) of the two bands, and therefore, when the evolution of free carriers is on comparable timescales, they are also difficult to disentangle. Furthermore, charge carrier localization at defect states cannot be unambiguously determined. To solve the latter, ultrafast sum-frequency generation with a white light continuum resonant with the in-gap defect states was implemented,^28^ reporting sub-picosecond cooling times of the electron in the CB.

Photoluminescence (PL) is sensitive to the DOS in the CB. In the ultrashort time domain, it can selectively detect the cooling of electrons down to the bottom of the band as well as the formation of free excitons.^27,29^ However, to our knowledge, nearly all time-resolved PL studies of TMOs have focused on the electron–hole recombination (see Table S1 of Refs. 28 and 30), which is on the tens of ps to ns time scales. This is, in particular, the case for the system of interest here, zinc oxide (ZnO). In this work, we implemented ultrafast PL upconversion spectroscopy in the UV in order to monitor the relaxation of electrons in the CB via the rise of the excitonic emission close to the bandgap (BG).

We complement the ultrafast PL study by ultrafast hard x-ray absorption spectroscopy (XAS). Over the past decade, time-resolved soft and hard XAS has increasingly been used to investigate the fate of charge carriers in photoexcited TMOs and perovskite nanoparticles (NPs) in colloidal solutions.^30–37^ In TMOs, the oxygen 2p-orbitals dominate the VB, while the metal 3d-orbitals dominate the CB.^38^ Therefore, the element-specificity of XAS implies to a certain extent, an electronic band selectivity, as was nicely illustrated in Ref. 37. In the case of TiO_2_,^31–34,37^ the ps and fs hard XAS studies showed signals that were predominantly due to changes of the Ti oxidation state from 4+ to 3+ and are, therefore, mostly sensitive to electron trapping. Ultrafast soft XAS at the O K-edge and the Ti L_2,3_-edges could simultaneously monitor both the hole and the electron trapping in photoexcited anatase TiO_2_ nanoparticles and single crystals.^37^

ZnO is a direct bandgap (3.4 eV) semiconductor that has a bulk exciton binding energy of 60 meV at room temperature, native n-type doping, and high conductivity,^1^ conferring to this material a high potential for optoelectronic applications in the visible and UV photon energy range. The band structure of ZnO exhibits a splitting of the top-most VB into three sub-bands usually termed as A, B, and C, due to a combination of crystal field and spin–orbit coupling.^39,40^ Transitions between these bands and the CB dominate the optical absorption spectrum (Fig. S1) at different polarizations. However, the band edge absorption stems from the vicinity of the VB maximum.^41,42^

Different to TiO_2_ where the electronic configuration of the metal atoms is d^0^, in ZnO, it is d^10^, and therefore, the metal atom cannot be reduced. Nevertheless, in a recent study of ZnO NPs photoexcited at 355 nm using time-resolved Zn K-edge XAS and x-ray emission spectroscopy (XES) with 80 ps resolution,^30^ dramatic changes were observed in the x-ray near-edge structure (XANES) spectra and the extended x-ray absorption fine structure (EXAFS) spectra. The time-resolved Zn K_α_ and K_β_ emission lines, which are sensitive to the electronic structure, showed, however, only weak charge density changes on the Zn atoms. This implied that the XANES and EXAFS spectral changes are largely due to structural effects. These spectral changes were rationalized by noting that ZnO is rich in singly charged Oxygen vacancies ( $O_{\text{vac}}^{+}$),^43^ and that upon photoexcitation, the free hole charge carriers generated in the VB of the material migrate and get trapped at the $O_{\text{vac}}^{+}$ defects to form doubly charged oxygen vacancies ( $O_{\text{vac}}^{++}$). Previously, theoretical calculations had shown that upon formation of a doubly charged oxygen vacancy,^44,45^ a dramatic increase in the $O_{\text{vac}}^{}-Zn$ distance occurs, displacing four Zn atoms per trapped hole charge. From the XANES and EXAFS features, the estimated $O_{\text{vac}}^{}-\mathrm{Zn}$ bond length increase was found to be ∼15% of its value prior to trapping.^30^ This result demonstrated the ability to detect hole trapping in ZnO by means of hard x-ray Zn K-edge absorption spectroscopy, even if the Zn atoms are not subject to significant electronic structure changes.

In order to disentangle the evolution of the electrons and holes in photoexcited ZnO, here we combine femtosecond-resolved UV PL upconversion^46^ studies with femtosecond-resolved Zn K-edge XANES. The UV PL study was carried out at the Lausanne Center for Ultrafast Science (LACUS) under 4.66 eV excitation, which is well above the optical BG of 3.4 eV (Fig. S1).^1^ The fs XANES measurements were carried out at the SACLA x-ray Free Electron Laser (XFEL) in Japan at an excitation energy of 3.49 eV, in order to minimize effects due to energetic electrons. Indeed, this energy is resonant with the blue wing of the first exciton and the red edge of the bandgap absorption (Fig. S1). In order to rationalize the x-ray results, we performed *ab initio* molecular dynamics (MD) simulations of the structural rearrangement around the newly formed doubly charged $O_{\text{vac}}^{}$. Details of the experimental and theoretical procedures and set-ups are given in the SI.

Our results show that electron cooling in the CB and formation of the exciton occur in <500 fs, in very good agreement with theoretical predictions,^41,42^ while the timescale for the hole response of ∼1.4 ps is governed by hole diffusion through the lattice and its trapping as the ensuing structural response is prompt according to the MD simulations.

## II. RESULTS

### A. Femtosecond photoluminescence studies

The steady-state PL spectrum of ZnO NPs at room temperature (Fig. S2) consists of a band around 3.37 eV and a broad band centered at ∼2.3 eV.^28,47^ The former is due to an excitonic electron–hole recombination between the CB and VB, while the latter has been attributed to recombination of CB electrons with hole defects (Oxygen vacancies) that form trap states within the bandgap.^28,30,47,48^ The time-resolved PL studies of RT ZnO in various forms (crystals, films, or nanoparticles) report different e-h recombination times for the excitonic and the green luminescence (Table S1 of Ref. 30), which, in addition, are sensitive to sample preparation.^28^

**FIG. 1.**
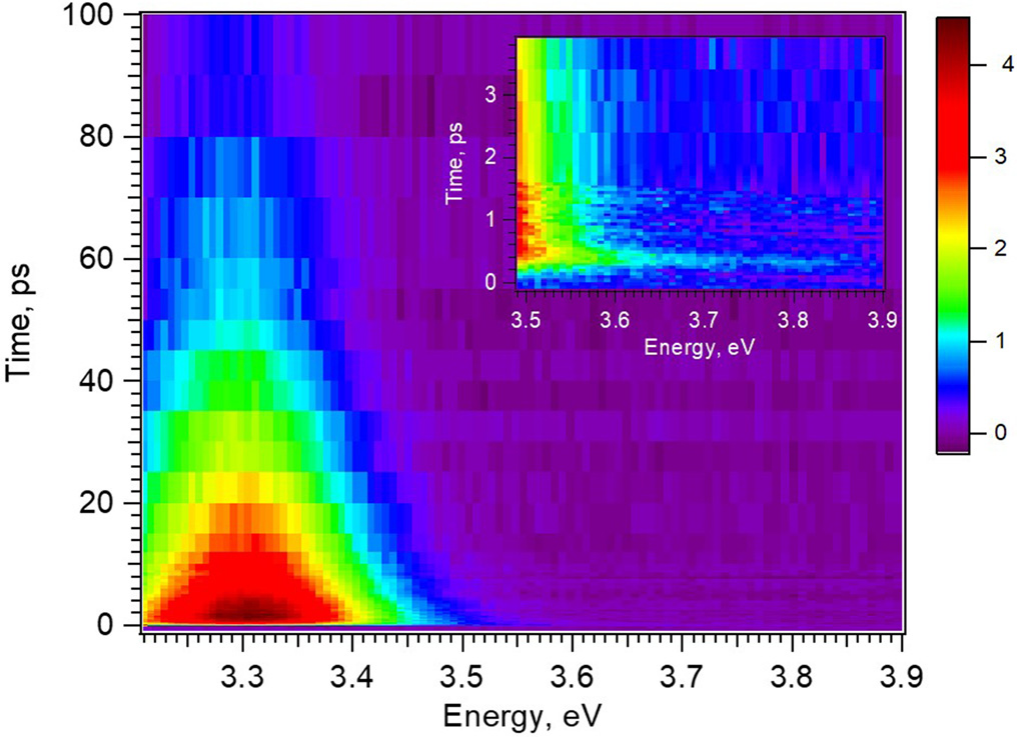
Time-energy (t–E) emission plot of the photoluminescence of ZnO nanoparticles in solution, measured upon excitation with 4.66 eV pulses of 5.4 mJ/cm^2^ fluence. The inset shows the high-energy side of the emission plot over the first 5 ps.

**FIG. 2.**
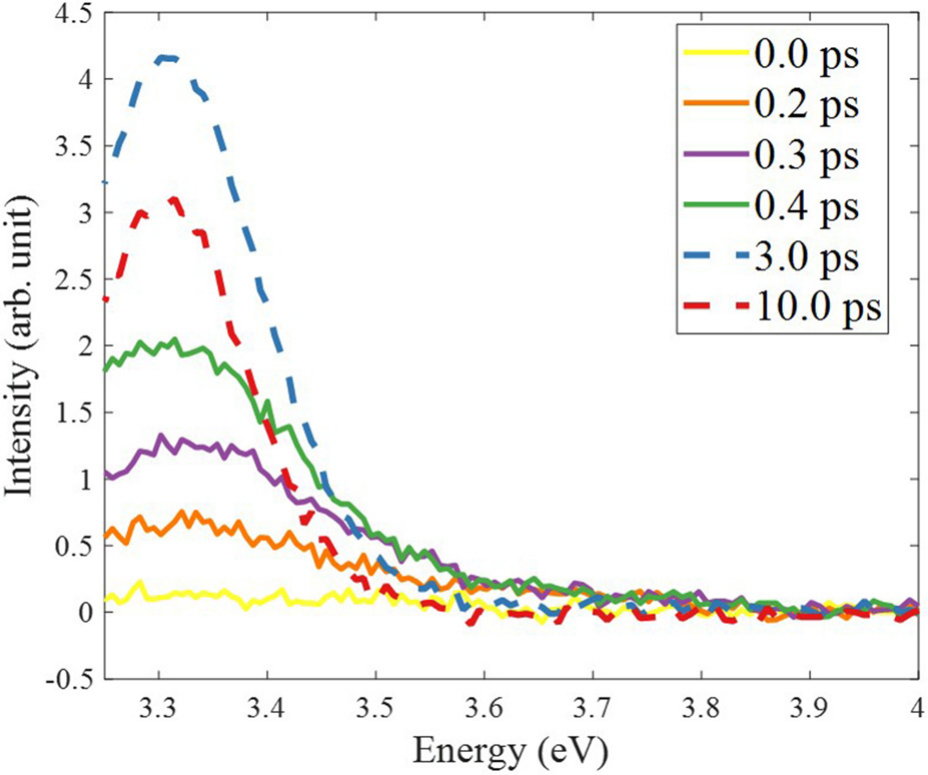
Photoluminescence spectra of ZnO nanoparticles in solution, at various time delay after excitation at 4.66 eV and at a fluence of 5.4 mJ/cm^2^. The spectra show the rise of the bandgap PL band at 3.31 eV. Note the relative contribution of the latter and the shoulder in the 3.45–3.7 eV region.

**FIG. 3.**
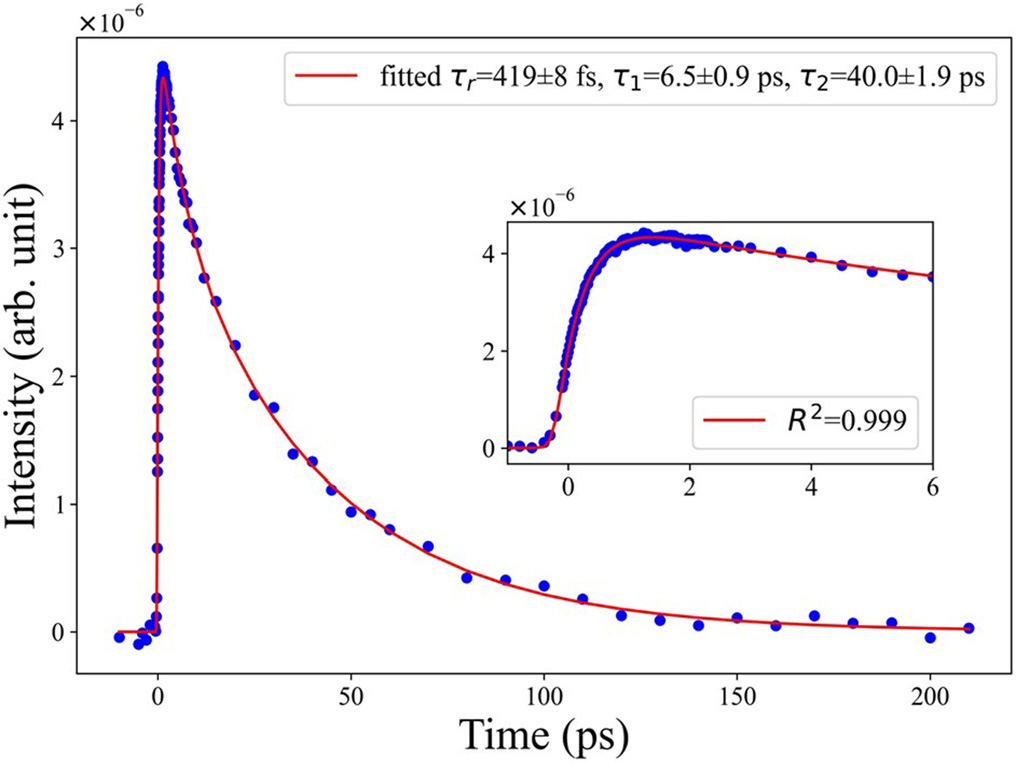
Kinetic traces over a time window of 200 ps of the photoluminescence of ZnO upon 266 nm excitation at a fluence of 5.4 mJ/cm^2^. The inset shows the time trace over a time window of 6 ps. The fits yield a rise time of approximately 420 fs and decay times of 6.5 and 40 ps.

Figure 1 shows a two-dimensional time-energy plot of the ZnO NP's excitonic PL over the first 100 ps after fs-excitation at 4.66 eV, while the inset zooms into the first 5 ps. The low-energy part of the emission is cut at 3.2 eV because of contamination by the strong scatter of the remnant 400 nm light from the laser. The high-energy part of the spectrum extends out to 3.9 eV; however, as the band edge is at ∼3.5 eV, this implies that any higher energy PL is in part reabsorbed by the sample. The remarkable feature here is that the PL is already at the energy of the exciton PL band from the earliest times, but the inset shows that the PL extends out to ∼3.8 eV, i.e., well above the gap, at the earliest times. Cuts of the time-energy plot at different time delays up to 10 ps are shown in Fig. 2. It can be seen that the emission grows within the first few hundreds of fs, at almost the same energy as the steady-state excitonic PL. The dependence of the PL intensity as a function of pump fluence is linear, as shown in Figs. S3–S5. In particular, the time traces recorded at the maximum (3.31 eV) of the PL are shown in Fig. S4 up to 5 ps for different fluences. Figure 3 shows the kinetic trace of the PL at the same energy for both long and short (insert) time windows. Figure S6 compares the time trace of the signal at early time with the Instrument response function (IRF), clearly showing that the signal's rise time is significantly longer. In Fig. 3, the long-time trace exhibits a biexponential behavior and it can be fitted with time constants of ∼6.5 and ∼40 ps (Table I). The fit of the short time traces convoluted with the IRF (approximated as a Gaussian) yields a value of ∼450 fs for the rise time, independent of the fluence (Table S1). Considering that the early time PL appears almost resonant with the steady-state one, this suggests that the rise time of the PL integrates the cooling time of the electrons in the CB as well as the formation of a relaxed excitonic state that yields the PL. With an excitation of 4.66 eV, i.e., 1.33 eV above the minimum of the CB, we can conclude that the electron cooling to the bottom of the CB occurs at a rate of approximately 3 meV/fs (1330 meV/450 fs). This is in excellent agreement with the predictions by Zhukov *et al.*^41^ that cooling of the high excess energy electrons is ultrafast, as they exploit the entire optical phonon phase space for energy dissipation.

**TABLE I.** Time constants (all entries are in ps) extracted from the present ultrafast near-UV PL and Zn K-edge XAS experiments and from the previous ps XAS experiment,^30^ and ultraviolet transient absorption (TA) spectroscopy.^49^ In the PL experiment, τ_r_ corresponds to electron cooling in the conduction band, while τ_1_ and τ_2_ reflect electron–hole recombination via the excitonic emission. In the fs-XAS experiment, τ_r_ corresponds to hole migration, trapping time, and the cage relaxation at the newly formed doubly charged vacancy. The longer times are due to electron–hole recombination via the trap PL in the green that is also reported in the ps-XAS experiment.^30^ Longer lifetimes have also been reported in the literature.^28,53^

Measurement	PL (4.66 eV)	Fs UV TA (4.2 eV) ^49^	fs-XAS (3.49 eV)	ps-XAS (3.49 eV) ^30^
τ_r_	0.42 ± 0.08	<0.15	1.4 ± 0.1	
τ_1_	6.5 ± 1	1.1		
τ_2_	40.0 ± 2			
τ_3_	⋯	88 ± 1	126 ± 44	200 ± 130
τ_4_		3900 ± 400		1200 ± 300

The steady-state PL spectrum of ZnO exhibits a rich fine structure with several lines, separated by a few tens of meV, attributed to different excitonic transitions,^1,28^ and it is, therefore, a composite band. The decay times of ∼6 ps and ∼40 ps may be due to different transitions therein and/or relaxation processes within the manifold of states giving rise to the PL.^28^ In Table I, we compare the times scales of the PL with those found using UV pump/UV continuum probe TA.^48^ In the latter case, the pump energy was 295 nm (4.20 eV), close to the present 4.66 eV excitation, and the probe was a continuum spanning the 280–360 nm range. The excitonic band was found to be bleached at t = 0, and its recovery timescales are given in Table I. While some of the time scales may bear a correspondence with the PL ones, it is difficult to be affirmative, as the TA is sensitive to the joint DOS of the VB and CB.

### B. Femtosecond Zn K-edge absorption spectroscopy

**FIG. 4.**
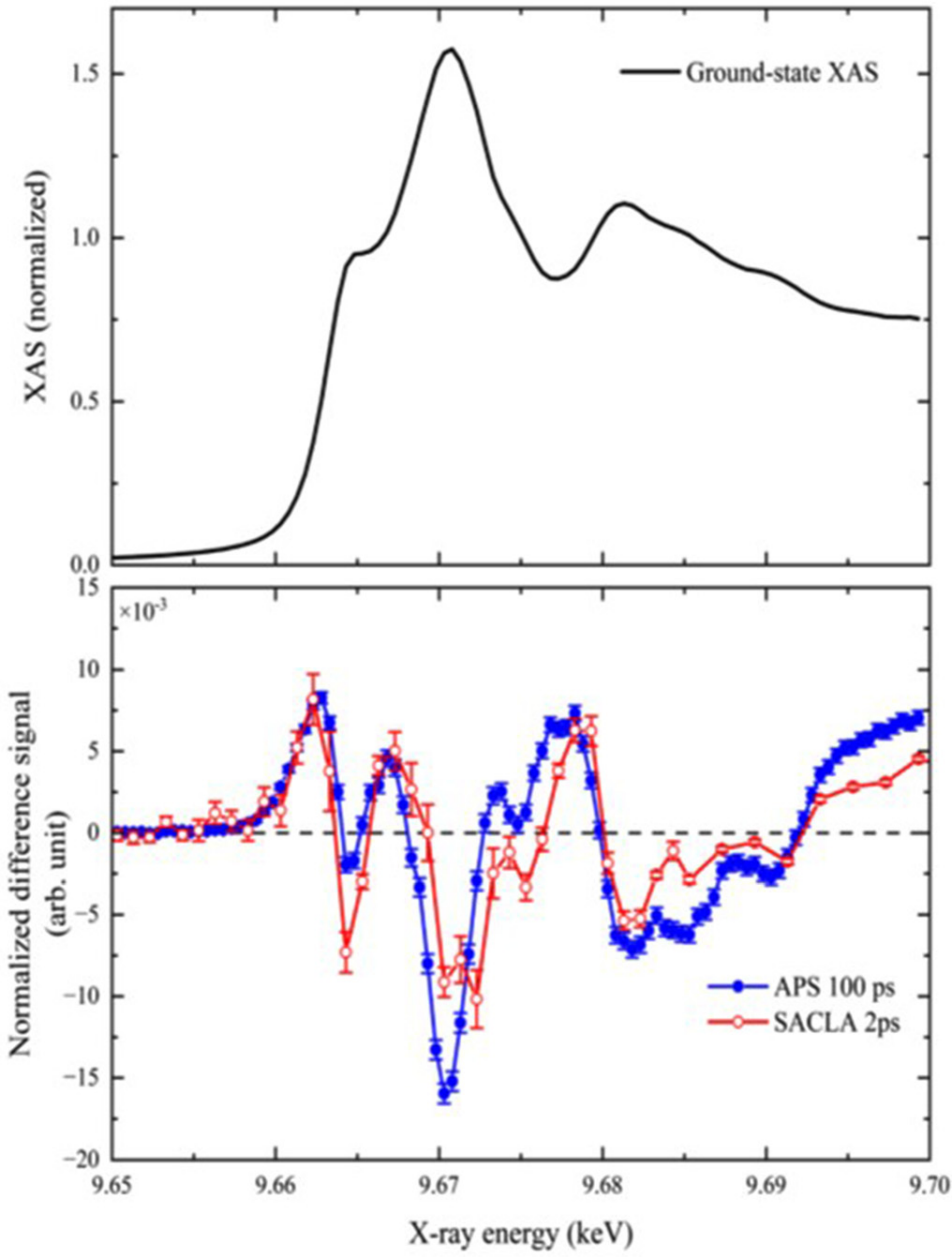
Steady-state Zn K-edge spectrum of ZnO nanoparticles in solution (black trace) along with the transient (laser-on minus laser-off) spectrum obtained in Ref. 30 using 80 ps resolution (blue points and trace) and the present femtosecond transient (red points and trace). The excitation wavelength for both the ps and the fs transient was 3.49 eV (355 nm).

**FIG. 5.**
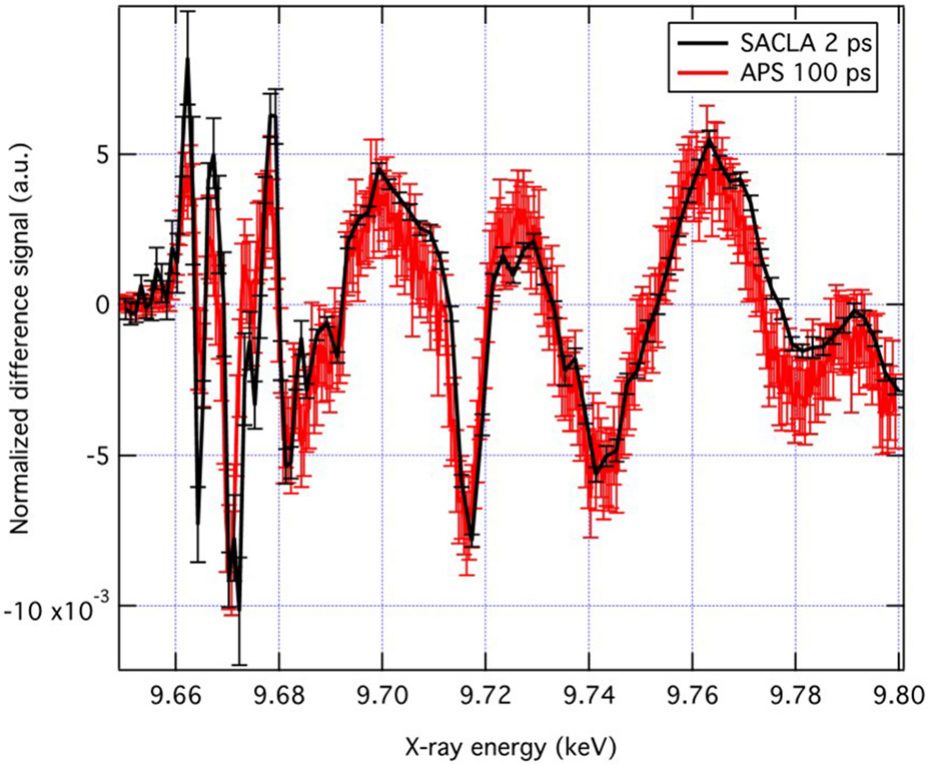
Transient x-ray absorption spectrum covering the XANES and EXAFS regions recorded at 100 ps time delay^30^ and at 2 ps time delay (this work).

Figure 4 shows the Zn K-edge XANES spectrum of ground state ZnO NPs (black trace) and the transient at 2 ps obtained upon 3.5 eV excitation, along with the transient spectrum previously obtained at 100 ps time delay.^30^ It can be seen that most of the features of the latter are already present in the 2 ps transient, but with somewhat different relative amplitudes. This implies that the most significant signatures of hole trapping, discussed in Ref. 30, are already present 2 ps after photoexcitation. Most of time-resolved XAS studies have focused on the XANES region as it provides more contrasted signals.^50–53^ However, one of the striking aspects of the ps-XAS study of ZnO^30^ is that the amplitude of the transient EXAFS was of comparable amplitude as the transient XANES. Figure 5 compares the entire transient XAS (XANES and EXAFS) spectra at 2 and 100 ps after bandgap photoexcitation. It can be seen that the two transients are quite similar both in the XANES and in the EXAFS regions. In Ref. 53, the transient linear dichroism XANES spectroscopy of epitaxial ZnO nanorods on monocrystalline quartz substrates was reported at 100 ps time delay. The similarities between transient and the temperature-induced XANES and EXAFS spectra led the authors not only to conclude that thermal effects are predominant but also to extract the actual electronic effects from their transients. Nevertheless, while not fully ruling out a thermal effect in the present transient XANES and EXAFS spectra, we do not hold it for predominant on the basis of the following reasons: a) the samples in Ref. 53 consisted of 1–2 *μ*m-long nanorods with a diameter of ∼70 nm. The nanorods did not have a fluid as an energy dissipation bath, and as pointed out by the authors, the long decay times (200–300 ns) of their transients were due to heat diffusion along the rods.^53^ Our samples consist of spherical NPs in solution, implying a more efficient heat dissipation; (b) the fact that the early time XANES and EXAFS reported here show such a resemblance with the 100 ps transient is quite remarkable and speaks against a predominance of thermal effects; (c) the band edge in the UV absorption of ZnO is very sensitive to temperature, and it undergoes a significant red shift with increasing temperature;^54^ however, the ultrafast TA probing across the bandgap shows an opposite trend, which again rules out a heating effect;^49^ (d) this is furthermore so that for the present x-ray measurements, the excess energy deposited to the system is minimized by the 3.49 eV excitation we used.

**FIG. 6.**
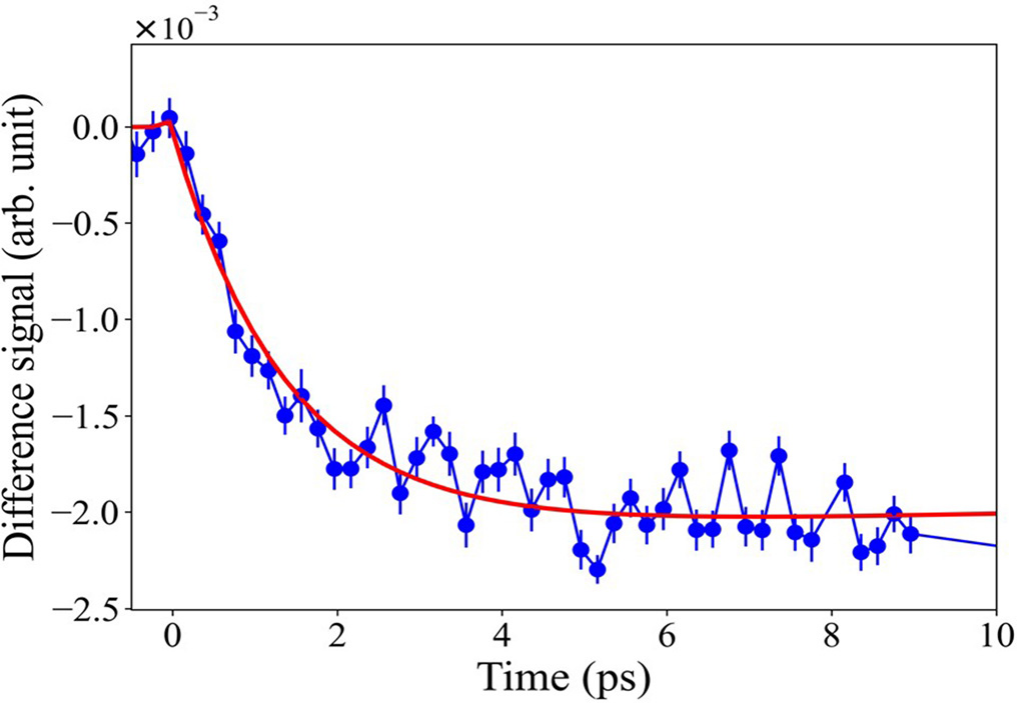
Time trace of the signal at 9.67 keV after excitation at 3.49 eV within the first 10 ps. Figure S7 shows the time traces and their fits at intermediate (up to 60 ps) and long (up to 200 ps) time delays.

The temporal evolution of the signal at 9.67 keV, where the amplitude of the x-ray transient is largest, is shown in Fig. 6 for early times, while Fig. S7 shows the kinetic traces at long and intermediate times. Figure 6 shows that the negative amplitude signal rises from zero, and it reaches a plateau by ∼5 ps, while the long-time trace (Fig. S7) shows a recovery that could be mono- or biexponential. We could satisfactorily fit the short- and long-time traces with a function consisting of a rising component and one decay component convoluted to the cross correlation of the experiment approximated as a Gaussian (see Sec. S2). The fit is shown in Figs. 6 and S7, and it yields a rising component of 1.4 ± 0.1 ps and a recovery one of 126 ± 44 ps. In the previous XAS study of ZnO NPs with 100 ps resolution,^30^ the kinetic trace at the same energy was scanned to longer delay times, and it exhibited a biexponential decay with time constants of 200 ± 130 ps and ∼1.2 ± 0.3 ns (Table I). The former is in the same scale as the recovery component reported here.

### C. *Ab initio* molecular dynamics simulations

The main focus of the present XAS measurement is the short component of ∼1.4 ps, which may reflect either a structural response ensuing an ultrafast trapping of the holes or the diffusion time of the holes in the lattice to the traps and their localization at the traps, followed by the latter's ensuing structural relaxation. In order to verify these hypotheses, we carried out *ab initio* molecular dynamics (MD) simulations. The details of the calculations are given in Sec. S3.

**FIG. 7.**
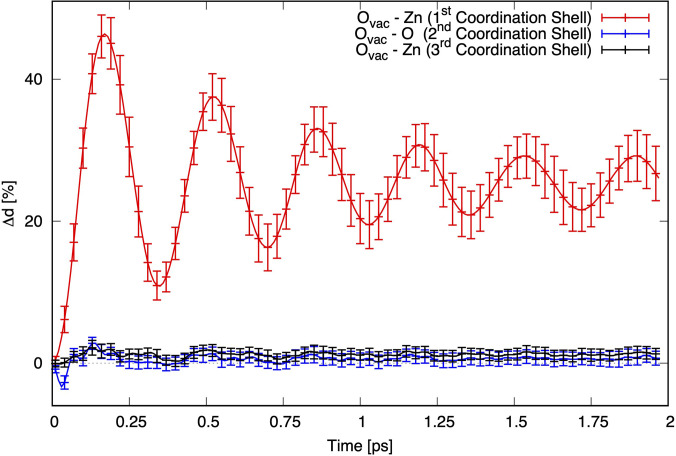
Percentage distance change from the oxygen vacancy to the first coordination shell composed of Zn atoms (red), the second coordination shell composed of O atoms (blue), and the third coordination shell composed of Zn atoms (black).

Figure 7 shows the percentage change (Δd) in the distance of the atoms from the oxygen vacancy upon impulsive switching of the vacancy from singly to doubly charged, which mimics a prompt hole trapping. The red trace shows the first coordination shell, i.e., the average of the distance between the Zn atoms around the oxygen vacancy upon hole trapping. It undergoes a rapid increase up to just over 40% in the first 200 fs, followed by an oscillatory behavior with a period around 0.33 ps that damps away in >1.5 ps. The blue/black trace shows the response of the second/third coordination shell, i.e.*,* the next-neighbor O/Zn shells around the vacancy. Clearly, only the first shell around the vacancy responds, while the next-neighbor shells do not, typical of an optical phonon. The value of the cage expansion converges to around 25%, which is close to original value of 23% calculated by Janotti and Van De Walle,^45^ while a value of 15% was extracted from the fit of the transient XANES at 100 ps.^30^ This deviation may be due to the fact that in the latter case, thermal effects had completely been neglected in the simulations of the transients. The oscillation period of the red trace in Fig. 7 is quite short (about 327–345 fs), and it most likely correspond to the E_2_ mode at 99 cm^−1^ (336 fs) reported in the Raman spectrum of ZnO and attributed to motion of Zn atoms.^55^ Its relatively long damping time is also in line with the narrow linewidth reported in the Raman spectrum. In addition, in PL spectra of low temperature ZnO, phonon replicas at this energy have been reported for the excitonic transition.^56^ This shows that the doubly charged oxygen vacancy has the characteristics of a small hole polaron.

In light of the aforementioned XAS and MD results, we discuss below the fate of holes in the system, after analyzing the optical ultrafast PL. The time constants extracted from the present ultrafast UV PL and fs-XAS studies are collected in Table I and are compared to those obtained in the previous ps-XAS^29^ and UV probe TA^49^ studies.

## III. DISCUSSION

The main focus of the present work is the early times of the evolution of charge carriers prior to the e-h radiative recombination. Regarding these times, a number of (mostly) UV-visible TA studies have been carried out on different types of ZnO: epitaxial thin films,^23^ various nanostructures (dots, rods, wires, and ribbons),^24,57^ ZnO/ZnMgO multiple quantum wells,^25^ molecular beam epitaxy films, and single crystals.^28^ These were generally carried out at RT and for different excitation fluences, and they concluded that the charge carrier relaxation spans timescales from 200 to 1000 fs. Ultrafast 2-photon photoemission studies of single crystals of ZnO excited at 4.19 eV were also carried out, reporting electron cooling times of 20–40 fs, followed by the formation of a surface exciton on a timescale of ∼200 fs.^58^ Considering that these studies used quite similar excitation energies, well above the bandgap, the fact that the values of the reported times are so scattered has to do with the pump fluence, the sample morphology, and, possibly, the environment.^24,57^

Using density functional theory (DFT) calculations, Zhukov and co-workers^41,42^ identified two regimes of electron-phonon cooling depending on the electron excess energy with respect to the highest phonon energy. At high electron excess energies, the whole phonon dispersion acts on the electron cooling, whose timescale spans from 100 to 500 fs, while below the cutoff of the highest energy phonon, only phonons with an energy lower than that of the electron excess energy will play a role in the cooling, and the energy loss time can span a very large range from sub-100 fs up to 10 ps with a rapid increase below an excess energy of 20 meV due to the reduction of the available phonon phase-space. For the holes,^41,42^ the energy loss time at any excess energy was found to be about three times smaller than the electron energy loss time.

In the PL experiment, τ_r_ in Table I corresponds to electron cooling in the conduction band and the formation of the exciton, in very good agreement with the aforementioned theoretical predictions.^41,42^ τ_1_ and τ_2_ reflect electron–hole recombination times via the excitonic emission. They are most probably due to decay of one of the many spectral components that makeup the excitonic emission^1,28^ or to a relaxation process within this same manifold. Finally, surely longer time components are present,^27,29^ but our scans are limited to 100 ps.

The present result of an electron cooling time of ∼450 fs is of importance for the description of electron injection times in dye-sensitized ZnO. Indeed, in contrast to dye-sensitized TiO_2_ where electron injection times are extremely short (<5 fs),^59,60^ in ZnO, the injection times are much longer, on the order of several tens of ps.^15,61–63^ The present results clearly confirm that they are entirely governed by the dye-ZnO interaction and not by the electron cooling within the ZnO substrate.^63^

Regarding the fs-XANES data, the ∼1.4 ps rise of the signal (Fig. 6) is to be contrasted with the prompt structural response found in the MD simulations (Fig. 7). We conclude from this that the former is mainly determined by the migration of holes, which then localize at singly charged oxygen vacancies that are expected to be more frequent near the surface of the NP due to a higher density of defects. In order to support this interpretation, we estimated the diffusion time of a hole inside the NP, assuming that it is created at its center.

Diffusivity (D) is related to charge mobility (*μ*) via^64^

$$D=\frac{kT}{q}\mu ,$$

where k is the Boltzmann constant, T is the temperature, and q = +e is the elementary charge. The diffusion time (τ) and length (L) are related via $L=\sqrt{D\tau }$.

In our case, the largest distance the hole travels in the (32 nm diameter) NP used in the fs-XAS experiment is 16 nm, assuming that the hole is created at the center of the NP. The values of the hole mobility of ZnO cover a very large range from 0.1 to 50 cm^2^/V/s, and taking *kT* = 25.4 meV at RT, we find that D varies from 0.002 54 to 1.27 cm^2^/s.^2^ This implies upper values of migration time of 2 ps to 1 ns. Considering the approximations made in this rough calculation, the fact that a distribution of distances is involved and the over 2–3 orders of magnitude uncertainty in the value of D, this estimate is quite satisfactory. In the context of our hypothesis, it would imply that the aforementioned upper value of D is closer to the real value.

The observation that the main features of the 2 ps XANES and EXAFS transients reproduce those of the 100 ps time delay measured at the synchrotron (Figs. 3 and 4) can be understood on the basis of Fig. 7, since the formation of a relaxed cage around the newly formed doubly charged $O_{\text{vac}}^{}$ takes less than 2 ps. In addition, one should stress that there is not only one category of defects and traps, and that the 100 ps transient, which was recorded with a 80 ps wide pulse,^30^ integrates a much larger sample of structural configurations of the doubly charged $O_{\text{vac}}^{}$'s.

Finally, it is important to stress that the fs-PL and fs-XAS do not monitor the same type of evolution. The ultrafast PL maps the energy relaxation of the electrons in the CB, while the fs-XAS maps the spatial migration of holes in the VB and their trapping. This may include hole energy relaxation, but the x-ray observable is not sensitive to it, since it only reports on structural changes at the $O_{\text{vac}}^{}$ s that trap the hole. In order to map the energy relaxation of holes, which, according to Zhukov *et al.*,^41,42^ is typically three times faster than the electron relaxation, one would need to detect the holes via ultrafast O K-edge XAS, as was recently reported for TiO_2_.^37^ Since the migration time of the holes is the rate determining step that governs their trapping, it is unlikely that phonon coherences similar to those found in the simulations could be generated. Given the predominance of this optical phonon mode, we should expect it in the steady-state PL spectra. However, as the mode is associated with traps, one would expect it in the visible (green) part of the spectrum (Fig. S2) since it is associated with hole traps.^30,47,48^ However, while the low temperature PL spectrum of ZnO shows rich fine structure of the UV bandgap PL, the green band is featureless.^56^ It would be exciting to investigate this phonon mode and its role in the polaron dynamics via impulsive stimulated Raman spectroscopy.

## IV. CONCLUSIONS

**FIG. 8.**
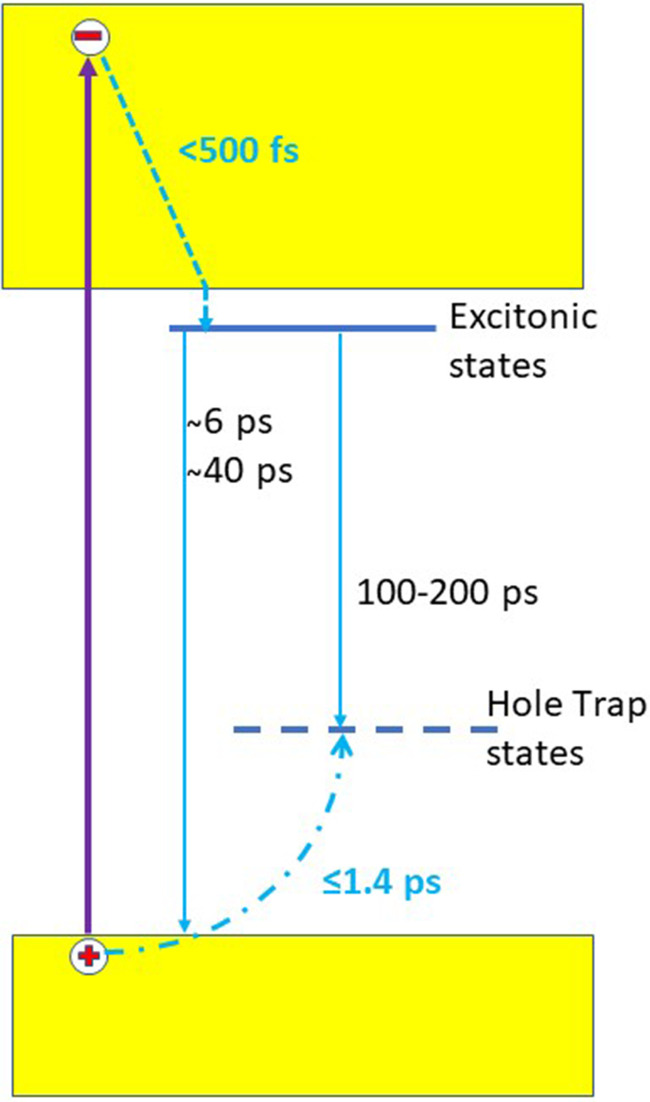
Scheme summarizing the results and showing the relaxation channels in ZnO for electrons and holes. The dashed line represents energy relaxation, while the dot-dashed line represents diffusion and trapping.

In summary, we presented a combined ultrafast UV photoluminescence and Zn K-edge absorption study of photoexcited ZnO nanoparticles in solution, complemented by *ab initio* molecular dynamics simulations. Our results show that electron cooling is ultrafast (<500 fs), in very good agreement with theoretical predictions.^41,42^ The fs x-ray absorption study shows that the signal grows on relatively slow time scales, but the transients at the first ps and at 100 ps are quite similar. In addition, *ab initio* molecular dynamics simulations show that upon hole trapping the Zn cage expansion around the doubly charged oxygen vacancy is prompt, and it stabilizes within about 2 ps. These results lead us to conclude that the ∼1.4 ps rise time of the Zn K-edge signal reflects the diffusion and trapping of holes after they have been created in the regular lattice. This scenario is supported by an estimate of the hole diffusion time in ZnO using literature values of the hole mobility. The processes investigated in this work are summarized in Fig. 8. The subsequent times found in both the optical PL, the fs-XAS experiments, and the deep-UV transient absorption studies are due to electron–hole recombination via both radiative and non-radiative mechanisms, but considering the complex nature of traps in ZnO and their variability with sample preparation, more studies are needed in order to attribute to specific processes.^65^

## Data Availability

The data that support the findings of this study are openly available in Zenodo at https://zenodo.org/record/8150479, Ref. 66.
